# Moving from theory to practice: experience of implementing a learning supporting model designed to increase patient involvement and autonomy in care

**DOI:** 10.1186/s13104-016-2165-5

**Published:** 2016-07-23

**Authors:** Rune Svanström, Susanne Andersson, Helena Rosén, Mia Berglund

**Affiliations:** School of Health and Education, University of Skövde, 54128 Skövde, Sweden; Health Sciences, University of Lund, Lund, Sweden

**Keywords:** Implementation, Learning supporting model, Nursing care, Person-centered care, Patient involvement, Reflection, Sweden

## Abstract

**Background:**

In implementing new programs of care, such as person-centered care, there is a risk that the focus will be at an organizational level, instead of a level that describes what happens in the personal development among staff. The aim of this study was to describe experiences of the implementation process of a learning supporting model designed to increase patient involvement and autonomy in care. The project, which lasted 2 years, involved training sessions, supervision and reflective meetings. Over the period, the staff who participated focused on developing their dialogues with patients to make the patients aware of their own capabilities and to encourage them to be fully involved in the treatment. A reflective lifeworld approach was used. Data were collected through interviews, notes and written stories, and analyzed using hermeneutic analysis with a focus on meanings.

**Results:**

At the beginning of the project, the participants perceived the model as abstract and difficult to understand but supervision and reflection sessions enabled understanding and changed the participants’ approach to caring. The participants described the model as an approach used in challenging patients to become involved in their care and to take charge of their lives when living with a chronic life-threatening disease. The participants’ experience of implementing the model has not been easy but has led to increased self-confidence and feelings of improved competence in dialogue with patients.

**Conclusions:**

Using the PARISH model when critically examining the results shows that in the implementation process there were some difficulties, e.g. the context was supportive and facilitating but there was no appointed facilitator. By making participation in improvement work voluntary, the impact of such work becomes less efficient, less cost-effective and probably less sustainable. Furthermore, implementation needs encouragement since changing approaches takes time and requires patience. Group supervision sessions seem an appropriate way to translate research into practice; systematic scheduled and mandatory group supervision sessions would, therefore, probably make implementation more robust and sustainable. In addition, a well-trained facilitator would be able to motivate staff to undertake daily reflection and participate in group supervision sessions. Reflection seems to be a key component in the personal learning necessary to change work routines and approaches.

## Background

In Sweden, the use of person-centered approaches has been shown [1] to improve care and increase patient participation. This type of approach requires that the patient is seen and understood as a unique individual [2] and their story is the core of their care [3]. The National Board on Health and Welfare has published national guidelines for care in cases of dementia. These emphasize person-centered care as a way to improve care for people with dementia [4], although at the time of publication, there was a shortage of scientific evidence in the area. A person-centered approach can improve healthcare providers’ understanding of the person with the disease instead of simply focusing on the diagnosis [3, 5]. Olsson [6] compared a person-centered approach with standard care after hip fracture and found that a patient-motivated accelerated training program based on the individual patient’s perceptions and motivation for rehabilitation resulted in shorter hospital stays, more successful rehabilitation and reduced costs. In implementing new programs of care, such as person-centered care, however, there is a risk that the focus will be at organizational level, instead of close to the patient, according to Ahgren [7]. This can be a problem if the task is to increase patient involvement in care [8]. Patient involvement can be understood as a way to support patients in their learning about their illness and help them to play an active role in their care and treatment [9].

Using research to increase patient involvement is a way to build evidence about care development, although the use of research in practice must be recognized as a complex and multifaceted process [5]. An awareness of research and evidence-based practice may help healthcare providers to ask relevant clinical questions about how to improve both the quality of care and clinical effectiveness [5]. Knowledge from research can be understood as a conceptual framework that informs policy, enhances understanding and may offer new perspectives on healthcare [10]. The context of healthcare seems to influence the use of research, as culture, leadership and evaluation of care with a strong focus on cost-effectiveness can be both facilitating and limiting. An efficient workplace culture contains elements such as a person-centered approach, transparent decision-making processes, and a learning culture [5]. Lorch [11] described how resistance and fear of change among staff can make it difficult to achieve educational goals and may limit the implementation of guidelines. Moreover, rapid turnover of staff causes other problems such as losing knowledge and disruption of collaboration. Habit, rather than a shortage of knowledge, skills, attitudes, or motivation may, however, be the main reason why healthcare professionals do not implement and adhere to best practice [12]. Kent et al. [13] highlighted the importance of understanding how research is translated into practice. According to Nilsen [14], there can be problems bringing together different professions’ ways of communicating, their values, perspectives on work and roles. It is, therefore, still questionable which methods and processes are most suitable for increasing the practice-related knowledge of healthcare staff.

Bick and Graham [15] argued that service users were now more involved in the design, content and evaluation of healthcare delivery. An example of patient involvement was work identifying core health domains for the assessment of rheumatoid arthritis. When patients were involved in the process, fatigue and general well-being were added as important outcomes [15]. Patients’ learning and education are important when developing a person-centered care approach that increases the patient’s ability to influence and participate, i.e., be involved in their own care [16]. In healthcare, the focus has long been on providing information and advice, not on supporting patients’ learning processes [17]. Getting used to illness takes time and is typically a struggle for patients [17, 18]. Previous research has shown that patients have often been given little or no support to transform the general knowledge presented by e.g. nurses and doctors into a personal understanding of how to live with disease [17, 19–21]. Patients who do not follow advice from healthcare professionals are often deemed to be noncompliant [22, 23]. A critical discussion of ethics related to strategies to enhance patients’ learning has thus become important [24]. The attitude that the patient is noncompliant may imply that unreflective healthcare professionals blame the patient [25, 26]. This may lead to difficulties for patients who wish to be involved in their care and treatment [17].

Information and education are often given on a superficial level, and patients are simply expected to do what they are told, which is contrary to person-centered care. Berglund [9] studied the learning of patients living with long-term illness and how that learning can be supported. This research suggests that genuine patient learning occurs on a deeper existential level and that there should be support for this. Turning points for learning and the importance of reflection have been identified, and based on this, a model that aims to support patients’ learning on an existential level has been developed [9, 27]. The model is based on a lifeworld approach [28–31]. Central to this model is the idea that nurses learn to apply a tactful and challenging approach based on the patient’s life situation, problems and issues (Table 1). The four theses in the model are aspects that the nurse and patient focus on during the dialogue. This is not a one-off occurrence; the intention is that the patient is to be more active during the continued dialogue that is resumed at every caring meeting between the patient and nurse. During the conversation, the nurse asks questions that support the reflection and help the patient verbalize his/her own situation, what it is like and possible ways to handle it. The intention is that the patient should feel his/her own power and see and use this to take charge in his/her own life situation and more directly express goals for the treatment and life.

**Table 1:** The four theses in the model “To take charge”

The challenge—to take charge of life with a long-term illness	
1.	Confronting the life situation and being challenged to make a change
2.	Positioning oneself at a distance when creating a new whole
3.	Developing self-awareness and moving from “one” to “I”
4.	Making learning visible to provide development and balance in life

In an earlier study [32] nurses’ experiences of supporting patient learning through this model were described.

### Aim

The aim of this study was to explore and describe experiences of the implementation process of a learning supporting model designed to increase patient involvement and autonomy in care.

The research questions were:

- How do staff experience implementation and use of the model in their everyday work?
- Are there new experiences?
- Does the caring attitude change?

## Methods

### Settings

The project of implementing the model in this study was initiated by the ward leaders of a hemodialysis center, but the crucial factor for implementing this model was a former patient’s view on the care at the center. In this patient’s experience, genuine dialogue involving questions that challenged the patient’s understanding and capability was absent. The patient contacted one of the researchers (MB) and departmental leaders, suggesting that nurses in the dialysis center should adopt the model. When the patient contacted the ward, the departmental leaders had recently read the research in MB’s thesis and were positive about the ideas. Furthermore, they also shared the patient’s view on care.

The hemodialysis center in a mid-sized hospital in southern Sweden consisted of two units, one for self-treatment dialysis and one for assisted hemodialysis (assHD). This project was designed for the assHD unit. The nursing staff with primary responsibility for the patients consisted of 11 registered nurses (RN), and one team leader (TL), who was the registered nurse in charge of the daily care work of the unit. The unit cared for 25–30 patients, who each attended the unit three to four times a week for sessions lasting 3–4 h each. Each RN had primary responsibility for the regular nursing care of two to four patients.

Participation in the study was voluntary, however, the implementation of the project and the study were carried out simultaneously; both were intertwined. The staff became affected by the project and the study whether they wanted to or not. The majority of RNs (n = 10) plus the TL agreed to participate in the study and they provided written consent. The department leaders were also invited to participate and consented. During the study, three RNs ended their employment and therefore their participation, while one of the three RNs who started working at the unit joined the study during its second and final year.

The implementation of the model into everyday work started with a session led by one of the researchers (MB) that focused on patients’ learning and the model. The aim of this session was to initiate a reflective process about the patients’ learning and the nurse’s role in supporting it among the participants. The participants were encouraged to focus on developing a dialogue with the patients to make them aware of their own capabilities and to encourage them to participate actively in their treatment. To initiate and to support the reflective process among the participants’, they wrote stories about their own experiences in supporting patients learning, group supervision sessions with MB were held four times per year. Monthly reflection meetings were also held. These meetings were arranged and led by one of the hospital’s reflection leaders (RLs).

### Research approach

Dahlberg, Drew and Nyström [33] developed a research approach, reflective lifeworld research, based on Giorgi’s [34] phenomenological approach. The overall aim of the reflective lifeworld approach is to describe and clarify lived experiences to increase knowledge of an individual’s personal experiences. Collecting lived experiences during the implementation period coincided with several activities that were carried out in order to implement the model. This meant that data were collected several times and in several ways. This way of implementing the model and collecting data helped to capture the nuances of any changes in experience and attitude among staff and aimed to capture as rich a description of the phenomenon as possible according to the lifeworld perspective [33]. Data were therefore collected through written stories and notes from the group supervision sessions and reflection meetings (Table 2). In order to deepen the understanding of the implementation of the model, qualitative open-ended interviews were added to the data collection (Table 2) [33, 34].

**Table 2:** Sources of data collected during the study

Data source	Number of collected data
Written stories: A situation when the participant felt successful (S) in supporting the patients’ learning A situation when the participant felt unsuccessful (NS) in supporting the patients’ learning	n = 30 Start of the project n = 12 stories; 7S/5NS In the middle of the project n = 4 stories; 4S The end of the project n = 14 stories; 8S/6NS
Notes from group supervision sessions	n = 7/planned 8
Notes from the monthly reflection meetings	n = 4/planned 18 more than four were carried out; no notes were taken from them
Open-ended interviews	n = 6 Interview 1: TL and department leader Interview 2: reflective leader Interview 3: two of the RNs (together) Interview 4: RN Interview 5: RN Interview 6: RN not participating in the project

### Data collection

During the project, one of the researchers (MB) wrote field notes. Written stories were collected from the participants who were asked to write two different stories on three occasions during the project (start, middle, and end). The stories were to include one story that described a situation when the participant felt successful in supporting the patients’ learning, and one that described a situation when the participant felt less successful in doing so. This was, at the same time, a part of the implementation and a way for the participants to reflect on their one role in the patient encounter (Table 2).

The group supervision sessions focused on the participants’ experiences of using the model when caring for patients. The sessions covered the challenges met, action taken to address these challenges and what the participants learned as a result. The notes written by the group supervisor after the sessions focused on the nurses’ reflections on their meetings with patients related to the learning approach, how this could be understood in light of the model, and what the participants had learned. These notes were helpful for the group supervisor in the continuous group supervision sessions, but were also used as data (Table 2).

Notes from the monthly reflection meetings were written by the reflection leader and focused on the subjects that had been discussed during the meetings, how the participants wanted to address the problem and what they had learned from it. These notes were helpful for the RL in the continuous group reflection process, but were also used as data (Table 2).

The open-ended interviews were carried out as a dialogue, to enable the participant to reflect on their own experiences. The dialogue focused on the participant’s experience of working with the model, participating in the project, and taking part in the reflection meetings and group supervision sessions. The interviews lasted 40 min on average and were digitally recorded and transcribed verbatim. They started with the invitation “Please tell me about your experiences of participating in this project”. Other questions and phrases used included “Tell me more”, “What do you mean when you say that?” and “What were your feelings?” (Table 2).

### Analysis

An inductive hermeneutic approach was used to analyze the data [33]. This type of analysis can be described as a continuous dialogue with the data, with a focus on meanings [33, 35], which in this study were the experience and attitudes of staff of the implementation. The analysis started with the initial whole of the data i.e., all the data were seen as one text. The researchers read the text and became familiar with the data. The focus of the reading then moved toward individual interviews, notes, field notes and stories, to identify the units of meaning in the data (i.e., a word, a sentence or a longer piece of text). The meaning units in the data were unpacked and each meaning was considered compared to the background of the whole, in an ongoing discussion among the researchers. The next phase involved the building and labeling of clusters of meanings. With the data and clusters of meaning as background, a new text was written and divided into themes. This was an ongoing, dynamic process among the researchers where both names, content and number of themes changed several times before ending in two themes. To validate the themes, quotations have been used to clarify the findings and exemplify the individual lived meanings. As the last step, a comprehensive interpretation was written with the themes, clusters of meaning and data as background, focusing on the changes in experience and attitudes during the implementation process. This interpretation is presented as a third theme.

### Ethical approval

The study was approved by the Ethical Committee in Gothenburg, Sweden (Dnr: 223-12). When presenting the results, we have tried to respect the confidentiality of the participants, but those who know where and when the project was carried out can probably identify single participants. To minimize the possibility of this, we have chosen not to attach the quotes to individual participants.

## Results

Staff experiences of implementing the model to increase patient involvement and autonomy in hemodialysis care are presented in three main themes: (1) Intent to change approach; (2) The importance of supervision and reflection; and (3) Changes achieved through the project.

### Intent to change the approach to care

The analysis revealed that there was a perception among the ward leaders and among some of the RNs that the care did not really give the patients enough support to be involved and to take responsibility for their treatment and health. This perception was confirmed by the former patient’s statement about care. This mutual impression of care made the ward leaders aware of the importance of mutual learning among staff in order to change the approach to care among them. They also acknowledged the importance of arranging meetings between researchers and staff to enable this learning. The ward leaders also recognized the fact that the aim and content of the project was in line with the hospital’s overall goal of working in a person-centered way, with one person saying:

*“This was something that we wanted to invest in, which we felt was along similar lines to earlier initiatives.”*

The desire to change the caring approach was articulated by the leaders, who saw potential in the interest expressed by the staff in developing new approaches to care, as well as the fact that there were resources available. In other words, “it was the right time” to implement a new approach. They believed that the unit offered unique opportunities for building this kind of caring relationship, since the nurses met the patients on a regular basis. There had already been several discussions in the unit about how to help the patients to make healthy changes in their daily lives:

*“We had a lot of discussions* [among staff] *about patients who had problems with, for example, weight* [in relation to fluid balance and kidney failure]*. We talked about how we as nurses should be able to reach out to the patient and engage in a good discussion about not gaining as much weight, so they don’t get this recurring problem, so to speak.”*

The willingness to change the caring approach was supported by the view so clearly expressed by the former patient. This patient believed that the research and the model developed through research would be useful for the nurses.

*“He would have liked someone to ask him these kinds of questions when he looked back on his experience as a patient in this unit.”*

One of the leaders said the following about the model and the willingness to alter the caring approach:

*“This is not lecturing, but it’s up to the patient to look inside himself and reflect on ‘how am I going to cope with this in a good way?’ So it doesn’t end up with me as a nurse saying, ‘you need to do this and that.’ That doesn’t usually work.”*

The model fits the need to increase communication with patients in a person-centered way. The leaders valued this and perceived the need for more knowledge and skills among the staff. They saw the model as consistent with patients’ requests to influence their own care, which places new demands on staff. They identified the model as congruent with their ideas about developing person-centered care in the unit:

*“Today’s society is based on knowledge and it is easy to get hold of information and knowledge, but we also have to offer other tools to manage this information.”*

Staff explained that their experience of participating in a former research project on empowerment had helped them to understand that they could not simply give the patients power, but that the way in which they, as nurses, acted could influence outcomes. The participants also suggested that the caring culture of the unit was unsupportive of patient empowerment. The main focus of staff was being responsible for the patients’ treatment but without really involving them. One of the participants gave an example of how she talked to the patients: “Today, I’ll take care of you, Anna. Don’t worry, just relax.” This attitude may be counterproductive for the patients’ health, because it does not provide patients with the tools to manage their health at home. There is an awareness of these difficulties, and the staff see empowerment and Motivational Interviewing (in which some of the nurses have been trained) as useful in helping patients to become more active in their treatment and to improve their well-being. In reaching this understanding, it was expressed that both staff and patients must change their views on the aims of care. This insight, and the ward leaders’ wish to use research in practice to change care, determined the use of the model.

A willingness to change the caring approach is crucial for the implementation of a new way of thinking and acting. This was revealed since some of the nurses did not participate very actively in the project. One of them stated that she was already competent in undertaking a caring dialogue with the patients. The leaders, however, were convinced that everyone needed to develop skills such as learning to ask the patients more questions. They were also aware of their failure to motivate the entire group. For example, one of the nurses who chose not to participate said that she felt that the project should have been preceded by a democratic process where the staff decided together about the project: “All of a sudden, we were just a part of it [the project]… I didn’t feel that it was ok.” The same nurse also found it difficult to imagine working from any models, saying, “No, this model … it is really hard for me to practice a model.”

The leaders were confident that the participating nurses were driven by curiosity, openness and a willingness to change their caring approach and actions. During the project, it became obvious that the participants saw their limitations and need for development. They all stated that they had learned to talk with patients in a new way and that reflection and learning together had been valuable for this.

### Importance of supervision and reflection

During the process of finding out how to carry out dialogues that were supportive of patient learning, the importance of supervision and reflection were highlighted by both the participants and the leaders. During the group supervision sessions, they reflected on their experience of dialogue with patients in relation to the model. Both their own and their colleagues’ actions, the needs of the patients, and the application of the model became obvious to the participants. One said: “I feel I had a Eureka moment.” She explained that suddenly, the differences between her goals and those of the patient had become obvious.

The participants said that the sessions had led to new approaches in their dialogue with patients. By being able to clarify various objectives, the participants established a better understanding between themselves and the patients. During the sessions, they discussed how their skill in directing the dialogue with patients had improved:

*“In general, we don’t ask explorative questions. Instead we are schooled to solve problems, so it’s kind of hard to change perspective.”*

Another participant said, “For me it has been an eye-opener.” By learning to ask questions, a different picture of the patient’s situation emerged. One question described as important was: “What are your expectations of me?” This question provided the opportunity to meet the patient on a new level, “more as a human being”, with their own goals and life in addition to the disease. During supervision, the participants began to understand that they needed to develop their faith in the patient’s inherent capabilities and find ways to communicate this belief to the patient.

During the group supervision sessions, the participants were coached in adopting a tactful attitude when meeting patients. They learned to meet the patient in the present moment, and start each dialogue by asking patients to talk about their current situation (i.e., here and now). This was experienced as an opportunity to challenge the patients’ understanding of the situation and the consequences of their own actions. The participants identified “the curious inquiring attitude” as being especially important. This could provide a shift in the dialogue, when the patients became aware of their significance as an individual and started talking about themselves as “I” instead of as “one” in general. During the group supervision sessions, the participants said that they had acquired the courage and tools to enable them to reflect and go deeper into the dialogue, challenging patients’ understanding and actions.

*“And something X taught me, which I have used a lot, is to support the patient’s self*-*confidence, and I have even found myself going from ‘one’ to ‘I’ when I talk…”*

As well as the group supervision sessions, the ambition was to have frequent reflection meetings. Many meetings were canceled, however, because of staff workloads. The group supervision sessions and the reflection meetings were scheduled but not mandatory for participants. Those who were working and could get there participated. As a result, the reflection meetings involved different people from one session to another, which the RL described as difficult:

*“Not everyone has been positive about attending but others have found it absolutely fantastic.”*

The RL described how, in the beginning, it was difficult for the participants to know what to bring to the meetings and reflect upon. This changed over time, as participants described how they had developed their abilities to reflect, express themselves, ask questions, and capture the small situations in everyday life as well as seeing the importance for patients’ learning.

The reflection meetings and the group supervision sessions gave the participants a sense of reciprocity; they shared experiences and learned from each other. The RL felt that it was an advantage that she was not involved in the patients’ care, which gave her the opportunity to ask naive questions. During the meetings, it became obvious how the project was influencing the participants, with one saying that she had started to reflect in a new way on her role as a nurse:

*“I am thinking about how to help the patient in their life more generally. Previously, I was so oriented on helping them with their treatment and doing it as well as possible but now it is about helping them to take charge of their own lives.”*

Through the written stories, it was possible to follow the participants’ progress and witness a more in-depth ability to reflect on their own role in particular situations. At the beginning of the project, the stories were about the control of blood sugar, phosphate, beverages, dietary guidelines, and the patients were perceived as passive recipients. The participants described how they tried to explain, inform, show test results and get help from other professionals to give the patient the best possible care, but “The patient was very negative and did not want to be told about the disease.” In the middle of the project, the stories became more about the patient’s fears, anxiety, anger, resistance and responsibility, instead of giving information to the patient. The participants wrote about how they involved the patients in their care:

*“The patient told me that last night she felt that her arm was cold. She thought she was going to die. During the next dialysis session, I listened and then asked if she had felt afraid of death.”*

In the final stories, the perception of the different goals of patients and participants was described, as well as how these differences affected relationships. The stories also described how the participants challenged unrealistic views or expectations among some patients, or confronted patients showing risky behavior, explaining how this behavior affected other patients in the unit.

In the stories, the participants described their choices, as well as discussing respect and expectations. These descriptions correspond to the learning supportive approach in the model and the intention to change approach and let the patient take charge of his/her own life. Group supervision sessions and reflection meetings were crucial to this change; the sessions facilitated the participants’ progress through reflection.

### Changes achieved through the project

At the beginning of the project, the model was perceived as abstract and difficult to understand. The supervision and reflection sessions were crucial for the participants as they enabled understanding (i.e., moving from theory to practice and increasing understanding of practice in relation to theory).

The participants’ experiences of dialogue with patients were the basis for the reflection meetings and group supervision sessions. During the group supervision sessions, the understanding of practice deepened as the meaning of the model became visible. The participants described the model as an approach used in challenging patients to become involved in their care and to take charge of their lives when living with a chronic life-threatening disease. The participants felt that this changed attitude in meetings with the patients seemed to support patients to participate in decisions about goals of care and appropriate measures. This required a change in practice among the participants, which in turn required support in the group supervision sessions.

Among the participants, there was a sense of disappointment which was also expressed by the leaders and the RL that not everyone joined the project and actively worked with the model. The leaders developed a consensus that the process was difficult, would take time, require supervision and need work on a long-term basis to achieve change. The model was perceived as a necessary tool to make care more patient-centered.

From an initial feeling that changing was a difficult task, the participants moved to understanding the model and embracing it during dialogue with patients and when reflecting on their experiences during supervision sessions. The experience of implementing the model has, from the staff’s perspective, not been easy but has led to increased self-confidence and feelings of deepened competence in dialogue with patients. Staff also perceived increased patient involvement and patient autonomy in hemodialysis care in the unit.

## Discussion

The findings demonstrate a willingness on the part of participants to make care more person-centered and increase patient involvement. Various projects had been initiated to increase the focus on this. The department leaders’ desire to change the caring approach was also in line with more general work at the hospital. This can, therefore, be seen as a good starting point for this project. The leaders’ awareness of research and evidence-based practice may have been of considerable help in motivating their staff to participate in the project [5]. Balfour and Clarke [36] suggest that developmental projects are often only anchored at the management level, but this project, following a former patient’s suggestion on improvement work at the unit, shows an admirable openness to change.

This study can only present a limited view on the implementation, as the perspectives of the patients were not included. Since the initiative to the implementation of the model came from the unit and there were time limits, there was no possibility to include the patients in the study. This is a limitation as there is no real evidence of change other than that expressed by the participants. However, carrying out this implementation without exploring the participants’ experience of the implementation of a new untested model would not have been ethical since there is a need to build evidence of its possible usefulness. Since the study was conducted, the model has been implemented in care work with elderly patients with chronic pain living at home, drawing upon the experiences from this study [37].

To adopt a critical perspective on this implementation project, the Promoting Action on Research Implementation in Health Services (PARIHS) model [38] has been used in the next section. According to this model, the success of the implementation of research depends on the interaction of evidence, context and facilitation. The degree of success thus depends on the relationships between the nature of the evidence and the context in which it will be implemented, as well as the mechanisms to facilitate change [38].

The model was developed through research involving patients with chronic illness and is grounded in patient experiences [9]. From a scientific perspective, evidence for the model has not yet been evaluated. This project was its first practical use. Despite the model not having an established evidence base, managers and users found it relevant in supporting their goal to involve patients in their care.

Evidence on patient education and learning is poor [16]. Our findings show a clear trend during the project; the participants stated that they developed competence in asking questions and capturing small situations to strengthen patients’ ability to be more involved in their care. At the end of the project, nurses also expressed a growing humility toward this task. The participants highlighted the importance of continuous supervision and reflection [28]. This suggests that the model gave them the courage and tools necessary to reach deeper into the dialogue with patients. This seems to be in line with the findings of earlier research [39] that reflection and supervision are useful when transferring knowledge into action and establishing change.

Context is the next point in the PARIHS model. In this study, the combination of leaders who are open to change and interested in using research, and a vision of the possibility of building caring relationships, can enable a sustainable change in terms of care. There was an active search for new ways of improving care. The unit can, therefore, be seen as a learning organization [10]. At the same time, participation was non-compulsory since the implementation was intertwined with research, which may have limited the outcomes. It also became obvious that not everyone agreed with the project. This raises questions about what happens during implementation of care improvement interventions, such as new hygiene routines or treatment regimens, if staff do not agree with the intervention, and simply do not comply. This puts great demands on units to create a transparent atmosphere, to remain a learning organization. Using the PARIHS model, the staff who did not participate in the project could be seen as not daring to leave the safety of their habitual way of working, although their position could also been seen as a healthy critical stance [10].

According to the PARIHS model, it is imperative that staff feel well informed and involved in decision-making when implementing a new approach to work. Our study confirms this. The individual’s responsibility to use research to improve their work on a day-to-day basis also has to be clear [10]. According to Hemsley-Brown [40], leadership plays a critical role in the implementation of new methods and models. In our study, the leaders felt it was important to use research in practice, and to participate in research projects. This was less important to the RNs, who simply wanted to develop their competence to support patients’ learning.

In this study, no-one was appointed to facilitate the implementation; this is something that Harvey et al. [41] and Rycroft-Malone [38] see as a key component. The TL shouldered the role because of her understanding of the model and she encouraged staff to attend the reflection meetings; she also took an active part in them. Furthermore, she followed up progress with the model during morning meetings, and used her personal work with the model as an example.

The role of the RL was more task-oriented. According to Harvey et al. [41], task-oriented facilitation is characterized by episodic contacts. In our study, only four of 18 planned meetings with the RL were documented. It is uncertain how many more of the planned meetings were carried out. One of the researchers (MB) met the participants at seven of eight planned group supervision sessions. There were 11 sets of notes from the reflection meetings and group supervision settings over 2 years, and it is questionable whether this was sufficient. These meetings encouraged the participants to work on the model. It would, therefore, have been valuable for either the RL or researcher to play a key role in the implementation [38], but this role fell instead on the TL, who participated in the meetings on the same terms as the others. Implementation is complex and takes time, especially when it is about changing approach, as in this study.

Rycroft-Malone et al. [42] concluded after an implementation study, that it is difficult to integrate even seemingly simple changes supported by strong evidence. The findings of our study suggest that implementation has led to positive effects for individual participants, which seem to increase their desire to stay involved, listening to patients’ existential questions, and also asking new kinds of questions and supporting the patients to take responsibility [c.f. 32]. However, this finding does not address whether there has been a positive effect on the unit overall. More studies are needed to establish the overall effects of the model, and on involving patients.

The conditions surrounding the implementation of the model were good. The hospital and the unit had explicit communicated goals and strategies for change. Potentially, if the concept had been anchored more clearly among the staff, it would probably increase the possibility of sustainable change. There seemed to be a clear trend among participants of increased ability to reflect and a growing skill to capture the small but important situations in meetings with patients. The implementation was probably also influenced by having no appointed facilitator. New research may demonstrate whether that is indeed the case.

One of the researchers (MB) took an active part in the implementation project when acting as the leader during supervision sessions. This entailed a risk of introducing bias but was a real opportunity to enable the implementation. By being involved, MB could capture the mood among staff and answer questions, but at the same time, she might get an overly positive impression of the implementation process. This was discussed among the researchers, with particular focus on our preconceptions on changing attitudes and actions and also about MB’s involvement in the unit. By bearing this in mind during analysis, we have tried to minimize its influence on the results.

## Conclusions

- When participation in improvement work is voluntary, the overall impact of such work may be less efficient, less cost-effective and probably less sustainable.
- Implementation needs to be supported on a long-term basis since changing approaches takes time and requires patience.
- Group supervision sessions are an appropriate means to translate research into practice.
- Systematic scheduled and mandatory group supervision sessions would probably help to make the implementation more robust and sustainable.
- Appointing a well-trained facilitator would help to motivate the staff in both their daily work and their reflections, ensuring that they were prepared to participate in the group supervision sessions.
- Reflection was a key component of the personal learning that is fundamental to changing work routines and approaches.
